# Application of an Efficient Enhancer in Gene Function Research

**DOI:** 10.3390/plants13223120

**Published:** 2024-11-06

**Authors:** Feng-Xian Guo, Rui-Xue Yang, Xia Yang, Jing Liu, Yin-Zheng Wang

**Affiliations:** 1 State Key Laboratory of Plant Diversity and Specialty Crops and Key Laboratory of Systematic and Evolutionary Botany, Institute of Botany, Chinese Academy of Sciences, Beijing 100093, China; guofengxian7@163.com (F.-X.G.); yangrx_98@163.com (R.-X.Y.); yangxia@ibcas.ac.cn (X.Y.); liujing2010@ibcas.ac.cn (J.L.); 2 China National Botanical Garden, Beijing 100093, China; 3 University of Chinese Academy of Sciences, Beijing 100049, China

**Keywords:** *Arabidopsis thaliana*, ARC, *Chirita pumila*, enhancer

## Abstract

Although great progress has been made in transgenic technology, increasing the expression level and thus promising the expected phenotypes of exogenous genes in transgenic plants is still a crucial task for genetic transformation and crop engineering. Here, we conducted a comparative study of the enhancing efficiency of three putative translational enhancers, including Ω (natural leader from a plant virus), *OsADH* 5′ (natural leader from a plant gene), and ARC (active ribosomal RNA complementary), using the transient gene expression systems of *Nicotiana benthamiana* and *Chirita pumila*. We demonstrate that three tandem repeats of ARC (3 × ARC) are more efficient than other enhancers in expression. The enhancing efficiency of 6 × ARC is further increased, up to 130 times the expression level without the insertion of enhancers. We further evaluated the enhancing efficiency of 6 × ARC under agrobacterium-mediated transformation systems. In *C. pumila*, 6 × ARC significantly amplifies the phenotypic effect of *CpCYC1* and *CpCYC2* in repressing stamen development and yellow pigmentation. In *Arabidopsis thaliana*, 6 × ARC and the *AtAP1* promoter work together to promote the accumulation of anthocyanin pigments in vegetative and reproductive organs. Most significantly, the fusion of 6 × ARC in a *CpCYC1/2* transgenic system in *C. pumila* fully reveals that these genes have the complete function of repressing the yellow spots, displaying an advantage in manifesting the function of exogenous genes. This study highlights the application potential of the enhancer 6 × ARC in gene function research in plants.

## 1. Introduction

Transgenic technology is widely used in various fields such as agricultural production and the biopharmaceutical industry, and especially in crop breeding. In transgenic plants, overexpressing target genes is often required to amplify phenotypes, and thus achieve crop ideotypes with high yields, high resistance to insecticides and herbicides, or high-value pharmaceutical products. Gene expression involves two biological processes: transcription (from DNA to RNA) and translation (from RNA to protein). Accordingly, strong promoters can be utilized to enhance gene expression at the transcriptional level [1,2], while specific promoters and translational enhancers can work together to enhance gene expression at the translational level [3,4]. CaMV 35S promoter [5], duplicated CaMV 35S promoter [6], maize polyubiquitin promoter [7], and rice actin promoter [8] are commonly used for the purpose of enhancing transcription. However, the global gene expression caused by these constitutive promoters can sometimes result in additional phenotypes, potentially complicating the analysis of gene function. Moreover, an excessive transcription of target genes has been known to trigger post-transcriptional gene silencing (PTGS), leading to unexpected phenotypes [9,10,11,12] or even opposite phenotypes deviating from expectations [13]. The joint action of specific promoters and translational enhancers is an efficient way to overcome the above-mentioned problems while enhancing gene expression at the translational level [3,4].

The 5′-untranslated regions (5′ UTR), derived from either plant virus RNA or plant genes, are vital to increase translational efficiency [14,15,16]. The commonly used translational enhancers from plant viruses include the 5′ UTR of tobacco mosaic virus (TMV) RNA [17], alfalfa mosaic virus (AMV) RNA [18], tobacco etch virus (TEV) RNA [19], and potato virus Y (PVY) RNA [20]. Notably, the Ω sequence derived from the 5′ UTR of TMV RNA has been widely used to enhance the translation of exogenous genes in transgenic plants and animals [21,22,23,24]. Among several translational enhancers derived from plant genes, the 5′ UTR of the alcohol dehydrogenase gene (*ADH* 5′ UTR) from *Arabidopsis thaliana* [25], *Nicotiana* [26], or *Oryza sativa* [25] has been successfully applied in various studies [26,27]. Regarding the actual effects in the application of these natural translational enhancers, researchers have found that they are not compatible with certain plant species [28]. For instance, the *AtADH* 5′ UTR and *NtADH* 5′ UTR can enhance the translational efficiency of mRNA in *A. thaliana* and *Nicotiana benthamiana*, but are ineffective in *O. sativa* [25]. Furthermore, some translational enhancers from plant virus are efficient in eudicots, but not in monocots, such as the 5′ UTR of TMV RNA and TEV RNA [28].

A large number of mRNAs contain segments that are complementary to the 18S rRNA within the 40S ribosomal subunit and thus increase the chance of mRNAs for translation [29]. ARC (active ribosomal RNA complementary) is such a sequence element that complements an internal 18S rRNA segment of rice and increases mRNA translational efficiency [30]. Moreover, two or more copies of ARC are much more efficient than a single copy, such as three copies of ARC (3 × ARC), exhibiting higher translational efficiency than Ω and the 5′ UTR of PVY RNA [30]. In addition, the region of 18S rRNA corresponding to ARC is absolutely conserved among rice, tobacco, maize, wheat, and human [30], hinting that ARC may have more extensive application than translational enhancers from plant virus RNA and plant genes. However, ARC is rarely used in transgenic plants [31]. Therefore, the translational efficiency of ARC in transgenic plants is not fully understood so far.

It is advisable to choose suitable model systems and appropriate genes for fully evaluating the application potential of ARC in transgenic plants. *Chirita pumila*, a member of the Gesneriaceae family belonging to Asteridae, has emerged as a new model plant owing to its high efficiency in both stable and transient genetic transformation systems [32,33,34]. A previous study indicates that two *CYCLOIDEA* (*CYC*)-like TCP genes in *C. pumila*, i.e., *CpCYC1* and *CpCYC2*, play an important role in controlling floral symmetry, floral orientation, and nectar guide patterning with differentiated overexpression phenotypes [35]. *A. thaliana* is a classical model plant belonging to Rosidae. In *A. thaliana*, *AtMYB75* plays a pivotal role in the accumulation of anthocyanin pigments and its overexpression caused obvious pigmentation in diverse plant tissues, including leaves, roots, stems, sepals and so on [36].

In this study, we first compared the translational efficiencies of Ω, *OsADH* 5′ UTR, and ARC using two transient gene expression systems, i.e., *N. benthamiana* and *C. pumila*. We found that the translational efficiency of 3 × ARC was much higher than that of Ω and *OsADH* 5′ UTR. When 6 × ARC was used, the translational efficiency of the reporter gene was further increased, up to 130 times the translation level without the insertion of enhancers. In *C. pumila*, the integration of 6 × ARC greatly enhanced the phenotypic effects of *CpCYC1* and *CpCYC2* in repressing stamen development and yellow pigmentation in both wild-type plants and *cyc1 cyc2* double-mutant. In *A. thaliana*, the application of 6 × ARC significantly promoted purple pigmentation in specific tissues. Significantly, the 6 × ARC translational enhancer can enable exogenous genes to fully manifest their functions in genetic transformation. These findings provide a technical basis for the wide application of 6 × ARC in gene function research.

## 2. Results

### 2.1. Translational Enhancer ARC Is More Efficient than Ω and OsADH 5′ UTR in Transient Gene Expression Assays

**Figure 1:**
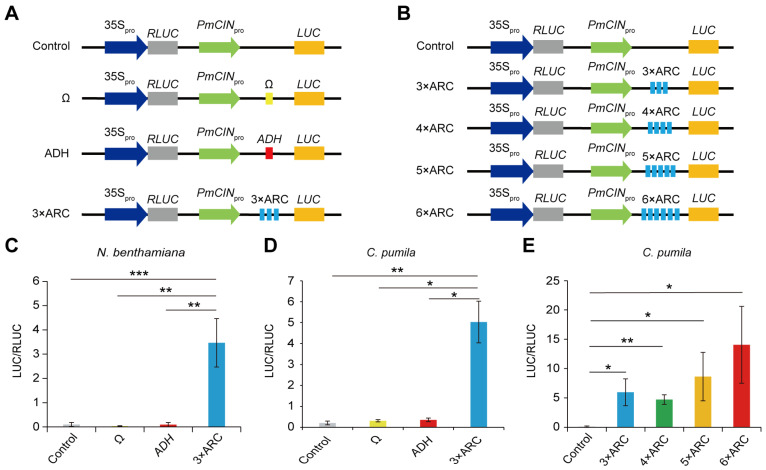
Transient gene expression assays of different translational enhancers in different plant systems. (**A**,**B**) The diagram showing the structure of plasmids harbouring different types of translational enhancers and different copy numbers of ARC. The firefly luciferase (*LUC*) gene was driven by the *PmCIN1* promoter. The plasmid with no translational enhancer was used as a control. Ω, 5′ UTR of tobacco mosaic virus (TMV) RNA; *ADH*, 5′ UTR of the alcohol dehydrogenase gene from *O. sativa*; ARC, active ribosomal RNA complementary. (**C**) Transient gene expression assays in *N. benthamiana* leaves. (**D**,**E**) Transient expression assays in *C. pumila* mesophyll protoplasts. Data shown were calculated from at least three independent replicates. Asterisks indicate significant differences between samples (Student’s *t*-test, * *p* < 0.05, ** *p* < 0.01, *** *p* < 0.001).

A previous study has demonstrated that 3 × ARC, an artificial translational enhancer complementary to 18S rRNA, greatly enhances the translational level of reporter genes in both the wheat germ cell-free system and *Orychophragmus violaceus* protoplasts, actually exceeding the Ω and 5′ UTR of PVY RNA derived from plant virus RNA [30]. In addition to the above-mentioned translational enhancers, those from plant genes, such as *ADH* 5′ UTR, can also enhance the translational efficiency of mRNAs [25]. Therefore, as the step to screen a high-efficient translational enhancer for transgenic experiments, we compared the translational activities of 3 × ARC, Ω, and *OsADH* 5′ UTR (Figure S1) in two distinct transient gene expression systems, i.e., *N. benthamiana* leaves and *C. pumila* mesophyll protoplasts. To this end, we constructed *PmCIN1_pro_-Ω:LUC*, *PmCIN1_pro_-ADH:LUC*, and *PmCIN1_pro_-3×ARC:LUC* plasmids in which the leaders were fused to the 5′ end of the *LUC* reporter gene under the control of the *PmCIN* promoter (Figure 1A). These plasmids included *35S_pro_:RLUC* as an internal control (Figure 1A). Given that *CINCINNATA*-like TCP genes are mainly expressed in leaves and petals [37,38], we used *PmCIN* promoter to activate the expression of the *LUC* gene in the leaves of *N. benthamiana* and *C. pumila*. The firefly luciferase (LUC) to renilla luciferase (RLUC) activity ratios showed that the artificial translational enhancer 3 × ARC exhibited significantly higher translational enhancing ability than two natural translational enhancers, i.e., Ω from plant virus and *OsADH* 5′ UTR from a plant gene in both *N. benthamiana* leaves and *C. pumila* mesophyll protoplasts (Figure 1C,D).

Since 3 × ARC results in a significant increase in the translational efficiency of the reporter gene in *N. benthamiana* and *C. pumila*, we hoped to test if multiple copies of ARC can lead to higher expression, as previously shown [30]. Therefore, we constructed *PmCIN1_pro_-4×ARC:LUC*, *PmCIN1_pro_-5×ARC:LUC*, and *PmCIN1_pro_-6×ARC:LUC* plasmids, in which 4 × ARC, 5 × ARC, and 6 × ARC were fused to the 5′ end of the *LUC* reporter gene under the control of the *PmCIN* promoter (Figure 1B and Figure S1). The results indicated that except for 4 × ARC, the increase in the copy number of ARC further enhanced the translational efficiency of the reporter gene, and 6 × ARC exhibited the highest translational efficiency, which was over 130 times higher than the control (Figure 1E).

In order to clarify whether 6 × ARC enhances gene expression at the translation level or at transcription and post-transcription levels, we transformed the reporter plasmids *PmCIN1pro:LUC* (control) and *PmCIN1pro-6×ARC:LUC* into *C. pumila* mesophyll protoplasts, respectively. The RNA level of *LUC* was detected using RT-qPCR, and the results (Figure S2) showed that there was no significant difference between *PmCIN1pro:LUC* (control) and *PmCIN1pro-6×ARC:LUC*. This result indicates that 6xARC is likely a translational enhancer.

Taken together, the efficiency of the artificial translational enhancer 3 × ARC is far superior to natural translational enhancers Ω and *OsADH* 5′ UTR in different plant systems, while 6 × ARC has the highest translational efficiency. However, it is unknown whether the 6 × ARC has actual effect in genetic transformation.

### 2.2. Transgenic Phenotypes of CpCYC1 Amplified by 6 × ARC

**Figure 2:**
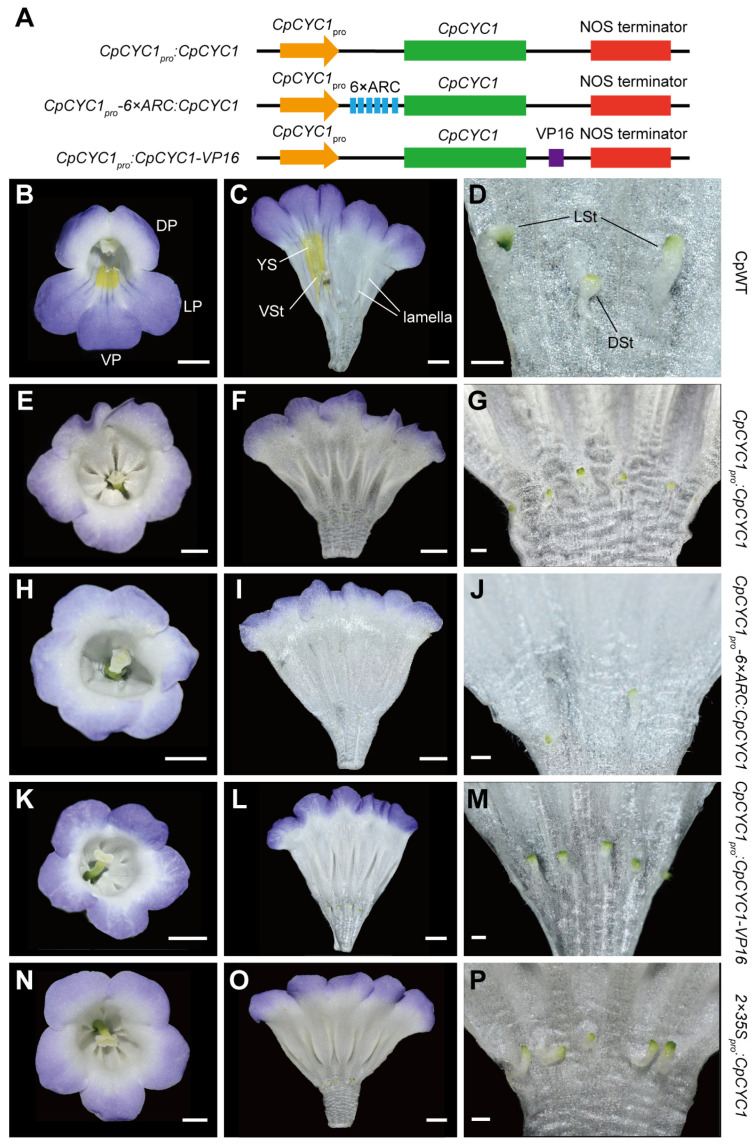
Translational enhancer 6 × ARC amplifies the function of *CpCYC1* in the wild-type plants of *C. pumila*. (**A**) The diagram showing the structure of different plasmids expressing *CpCYC1* gene. (**B**–**D**) Flowers of wild-type (WT) plants. (**B**) Front view of a wild-type flower showing dorsal/lateral/ventral (DP/LP/VP) petals. Scale bar: 0.5 cm. (**C**,**D**) The inner structure of wild-type flowers showing yellow spots (YS), dorsal/lateral/ventral stamens (DSt/LSt/VSt), and two dorsal lamellae structures. Scale bars: (**C**) 0.5 cm; (**D**) 0.1 cm. (**E**–**G**) Flowers of *CpCYC1_pro_:CpCYC1*. Scale bars: (**E**,**F**) 0.5 cm; (**G**) 0.1 cm. (**H**–**J**) Flowers of *CpCYC1_pro_-6×ARC:CpCYC1*. Scale bars: (**H**,**I**) 0.5 cm; (**J**) 0.1 cm. (**K**–**M**) Flowers of *CpCYC1_pro_:CpCYC1-VP16*. Scale bars: (**K**,**L**) 0.5 cm; (**M**) 0.1 cm. (**N**–**P**) Flowers of *2×35S_pro_:CpCYC1* was used as a control, adapted from [35]. Scale bars: (**N**,**O**) 0.5 cm; (**P**) 0.1 cm.

To evaluate the effect of 6 × ARC in transgenic research, we selected *CpCYC1*, belonging to the TCP transcription factor family, as a candidate gene because its overexpression in *C. pumila* has obvious phenotypes in floral symmetry, floral orientation, and nectar guide patterning [35]. We constructed *CpCYC1_pro_:CpCYC1* and *CpCYC1_pro_-6×ARC:CpCYC1* plasmids (Figure 2A) and transferred them into the wild-type plants of *C. pumila*. Among 18 and 32 independent transgenic lines of *CpCYC1_pro_:CpCYC1* and *CpCYC1_pro_-6×ARC:CpCYC1*, 5 and 21 lines generated evidently different flower phenotypes from wild-type plants, respectively (Figure 2 and Table 1). *C. pumila* has typical zygomorphic flowers mainly reflected in the second and third whorls (Figure 2B,C). The second whorl consists of three different types of petals, including two dorsal petals, two lateral petals, and one ventral petal (Figure 2B). In addition to the difference in morphology and size, yellow spots near the throat of ventral corolla tube and a structure of two lamellae inside the dorsal corolla tube are also the characteristics of zygomorphic flowers of *C. pumila* (Figure 2B,C). In the third whorl, one dorsal and two lateral stamens are sterile, and two ventral stamens are fertile (Figure 2C,D). Different from wild-type plants, *CpCYC1_pro_:CpCYC1* transgenic lines produced dorsalized actinomorphic flowers in which all petals adopted the morphology of the dorsal petals, the dorsal lamella structure expanded into all regions of the corolla tube, while yellow spots disappeared (Figure 2E–G). In the third whorl, all five stamens became sterile (Figure 2G), similar to the dorsal staminode in wild-type flowers (Figure 2D). *CpCYC1_pro_-6×ARC:CpCYC1* transgenic lines also produced dorsalized actinomorphic flowers (Figure 2H–J). However, the development of stamens in *CpCYC1_pro_-6×ARC:CpCYC1* flowers was severely repressed so that some of them completely disappeared (Figure 2J). On the contrary, all stamens of *CpCYC1_pro_:CpCYC1* were tiny but still visible (Figure 2G).

VP16 is a transcriptional activator domain which can amplify exogenous gene phenotype by strongly activating downstream target genes [39,40]. Therefore, we also constructed *CpCYC1_pro_:CpCYC1-VP16* plasmid (Figure 2A) and transferred it into the wild-type plants of *C. pumila*. We then compared the flower phenotypes of *CpCYC1*_pro_*-6×ARC*:*CpCYC1* with those of *CpCYC1*_pro_:*CpCYC1-VP16* and *2×35S_pro_:CpCYC1* described before [35]. The results showed that *CpCYC1_pro_:CpCYC1-VP16* (Figure 2K–M) generated similar dorsalized actinomorphic flowers to *CpCYC1_pro_:CpCYC1* (Figure 2E–G) and *2×35S_pro_:CpCYC1* (Figure 2N–P). In all these transgenic lines, five stamens were sterile but still visible (Figure 2G,M,P). Taken together, 6 × ARC was most efficient in amplifying the phenotypic effect of *CpCYC1* gene in repressing stamen development.

**Figure 3:**
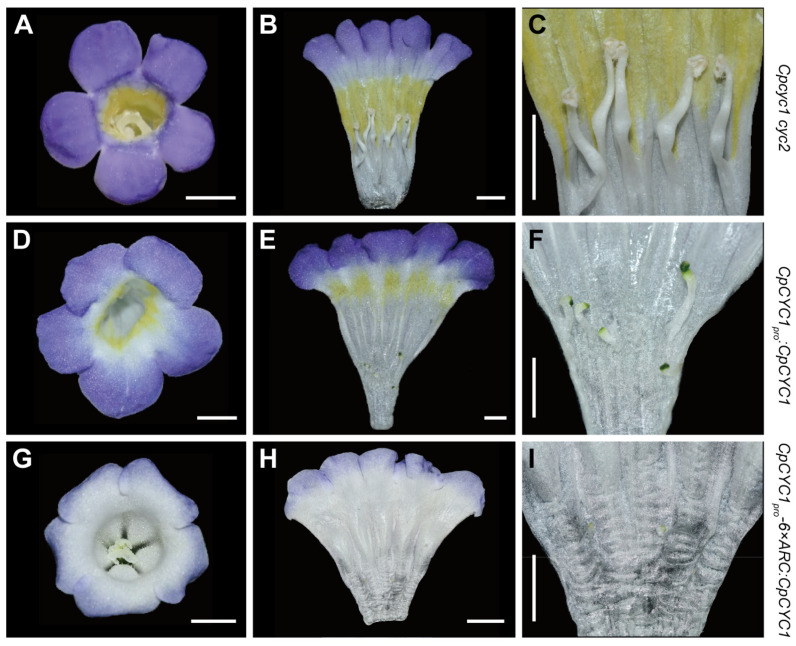
Translational enhancer 6 × ARC amplifies the function of *CpCYC1* in the *cyc1 cyc2* double-mutant of *C. pumila*. (**A**–**C**) Flowers of the *cyc1 cyc2* double-mutant. (**D**–**F**) Flowers of *CpCYC1_pro_:CpCYC1*. (**G**–**I**) Flowers of *CpCYC1_pro_-6×ARC:CpCYC1*. Scale bars: 0.5 cm.

Given that the transformation of *CpCYC1_pro_-6×ARC:CpCYC1* plasmid completely repressed the development of stamens, we next wanted to know whether it had a similar function in *cyc1 cyc2* double-mutant of *C. pumila* with all stamens fertile [35]. Therefore, we transferred the *CpCYC1_pro_-6×ARC:CpCYC1* plasmid (Figure 2A) into the *cyc1 cyc2* double-mutant. Only *CpCYC1_pro_:CpCYC1* was transformed as a control because *CpCYC1_pro_:CpCYC1-VP16* and *2×35S_pro_:CpCYC1* have similar phenotypes to *CpCYC1_pro_:CpCYC1* in wild-type plants. According to [35], the *cyc1 cyc2* double-mutant generated by CRISPR/Cas9-mediated gene editing exhibits ventralized actinomorphic flowers in which all petals adopt the identity of ventral petals with yellow spots expanded into all regions of the corolla tube, and all stamens become fertile (Figure 3A–C). In *CpCYC1_pro_:CpCYC1* flowers, floral symmetry changed from ventralized to partially dorsalized actinomorphy in both the second and third whorls (Figure 3D–F). In the second whorl, even though all petal lobes adopted the morphology of dorsal petals and the lamella structures were ectopically produced, yellow spots did not completely disappear but were restricted to the throat of the corolla tube (Figure 3D,E). In the third whorl, five stamens were sterile but still clearly visible (Figure 3F). In contrast, the flowers of *CpCYC1_pro_-6×ARC:CpCYC1* were completely dorsalized actinomorphic in both the second and third whorls with complete disappearance of yellow pigmentation and almost the loss of stamens (Figure 3G–I).

Taken together, the application of the translational enhancer 6 × ARC significantly amplified the role of the *CpCYC1* gene in inhibiting stamen development and repressing yellow pigmentation in both the wild-type and *cyc1 cyc2* double-mutant of *C. pumila*. However, the transformation of both *CpCYC1_pro_:CpCYC1* and *CpCYC1_pro_-6×ARC:CpCYC1* generated dorsalized actinomorphic flowers, similar to those of *2×35S_pro_:CpCYC1* [35], in both genetic backgrounds in this study. Further research might be crucial to determine whether the combined action of 6 × ARC and a longer promoter of *CpCYC1* could restore the *cyc1 cyc2* double-mutant to wild-type plants.

A previous study has demonstrated that *CpCYC1* can positively regulate its own expression [35]. This suggests that an increase in the transcriptional level of the *CpCYC1* gene may indirectly reflect its enhanced translational efficiency by 6 × ARC. Consequently, we detected the expression of *CpCYC1* at the transcriptional level using RT-qPCR with a lack of CpCYC1 antibody. Consistent with a previous report [35], the *CpCYC1* gene was specifically expressed in dorsal petals in wild-type plants (Figure S3A). In *CpCYC1_pro_:CpCYC1* plants, the *CpCYC1* gene was highly expressed in all petals (Figure S3A), hinting the non-specificity of the promoter. Importantly, RT-qPCR results showed that the expression level of *CpCYC1* in *CpCYC1_pro_-6×ARC:CpCYC1* was significantly higher than in *CpCYC1_pro_:CpCYC1* under the wild-type transgenic background (Figure S3A). A similar phenomenon was also found in the transgenic background of the *cyc1 cyc2* double-mutant (Figure S3B). In addition, with the wild-type transgenic background, the expression level of *CpCYC1* in *CpCYC1_pro_-6×ARC:CpCYC1* was also higher than in *CpCYC1_pro_:CpCYC1-VP16* (Figure S3A). Taken together, the translational enhancer 6 × ARC significantly increased the expression level of *CpCYC1*.

### 2.3. Transgenic Phenotypes of CpCYC2 with the Fusion of 6 × ARC

**Figure 4:**
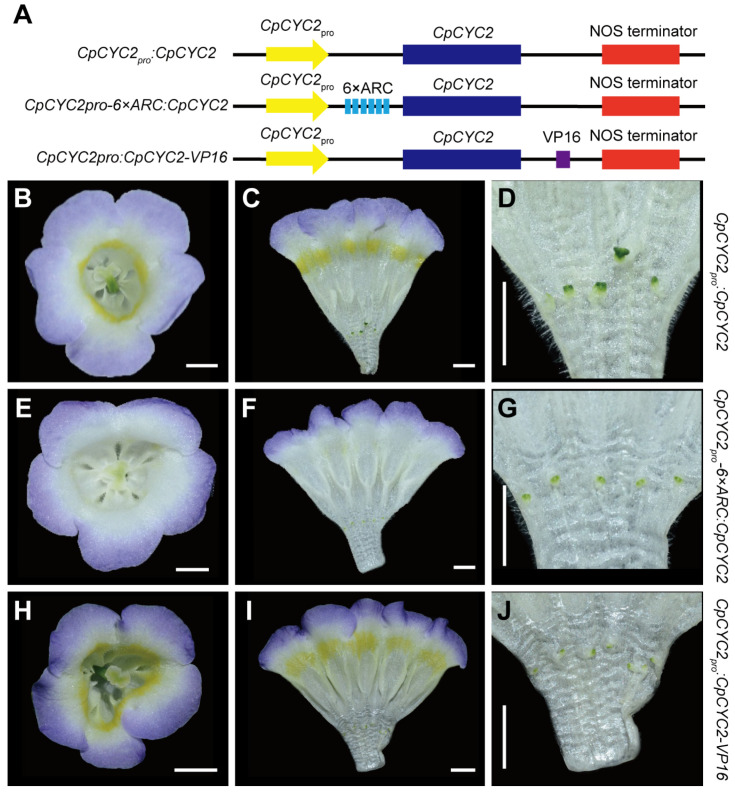
Translational enhancer 6 × ARC amplifies the function of *CpCYC2* in the *cyc1 cyc2* double-mutant of *C. pumila*. (**A**) The diagram showing the structure of plasmids expressing the *CpCYC2* gene. (**B**–**D**) The flowers of *CpCYC2_pro_:CpCYC2*. (**E**–**G**) The flowers of *CpCYC2_pro_-6×ARC:CpCYC2*. (**H**–**J**) The flowers of *CpCYC2_pro_:CpCYC2-VP16*. Scale bars: 0.5 cm.

In *C. pumila*, another *CYC*-like TCP gene *CpCYC2* also controls floral symmetry and nectar guide patterning even though it has a narrower expression domain and a much lower expression level than *CpCYC1* [35]. To investigate whether the integration of 6 × ARC can also amplify the function of *CpCYC2*, we constructed *CpCYC2_pro_:CpCYC2*, *CpCYC2_pro_-6×ARC:CpCYC2*, and *CpCYC2_pro_:CpCYC2-VP16* plasmids (Figure 4A). As described above, the phenotype difference between *CpCYC1_pro_-6×ARC:CpCYC1* and other constructs was more conspicuous in the double-mutant than in wild-type plants (comparing Figure 2 with Figure 3). Therefore, the three constructs related to *CpCYC2* were only transformed into the *cyc1 cyc2* double-mutant.

The results showed that the transformation of all the three constructs in the double-mutant background produced dorsalized actinomorphic flowers in which all stamens became sterile (Figure 4B–J). However, *CpCYC2_pro_:CpCYC2*, *CpCYC2_pro_-6×ARC:CpCYC2*, and *CpCYC2_pro_:CpCYC2-VP16* flowers were different in both the second and third whorls. In the second whorl, the yellow spots of both *CpCYC2_pro_:CpCYC2* and *CpCYC2_pro_:CpCYC2-VP16* flowers were locally distributed near the throat of the corolla tube, while they were completely disappeared from *CpCYC2_pro_-6×ARC:CpCYC2* flowers (Figure 4C,F,I). In the third whorl, the stamens of both *CpCYC2_pro_-6×ARC:CpCYC2* and *CpCYC2_pro_:CpCYC2-VP16* were much smaller than those of *CpCYC2_pro_:CpCYC2* (Figure 4D,G,J). Therefore, the application of 6 × ARC also amplified the function of *CpCYC2* gene in inhibiting stamen development and repressing yellow pigmentation.

It should be noted that the stamens of *CpCYC2_pro_-6×ARC:CpCYC2* were tiny but visible, different from *CpCYC1_pro_-6×ARC:CpCYC1* flowers, confirming again that *CpCYC1* plays a major role while *CpCYC2* plays a minor role in floral zygomorphy in *C. pumila* [35]. In addition, *CpCYC2_pro_:CpCYC2* transgenic lines did not restore the phenotype of the *cyc1 cyc2* double-mutant but produced dorsalized actinomorphic flowers similar to those of *2×35S_pro_:CpCYC2* [35], hinting the failure of the 1.7 Kb promoter in mimicking the native expression pattern of the *CpCYC2* gene.

### 2.4. Phenotype Frequency of Transformation with the Fusion of 6 × ARC in C. pumila

In addition to amplifying exogenous gene phenotype, we wondered if the translational enhancer 6 × ARC is more efficient to increase the phenotype frequency. Thus, we calculated the phenotype frequency that is defined as the ratio between the number of transgenic plants with expected phenotypes and the number of positive transgenic plants. In the wild-type genetic background, we obtained 18 *CpCYC1*_Pro_:*CpCYC1* positive transgenic plants, and 5 of them showed obvious phenotypes, with a phenotype frequency of 27.78% (Table 1). For the *CpCYC1_Pro_:CpCYC1-VP16* transgenic plants, the phenotype frequency was 36.36% (Table 1). Among 32 *CpCYC1_Pro_-6×ARC:CpCYC1* positive transgenic plants, 21 displayed obvious phenotypes with a phenotype frequency of 65.63%, much higher than both *CpCYC1_Pro_:CpCYC1* and *CpCYC1_Pro_:CpCYC1-VP16* (Table 1).

In the *cyc1 cyc2* double-mutant background, the phenotype frequency of *CpCYC1_Pro_-6×ARC:CpCYC1* was 51.85%, much higher than that of *CpCYC1_Pro_:CpCYC1* (28.57%). A similar result was also found in the *CpCYC2* gene in which the phenotype frequency of *CpCYC2_Pro_-6×ARC:CpCYC2* was 50%, while those of *CpCYC2*_Pro_:*CpCYC2* and *CpCYC2_Pro_:CpCYC2-VP16* were 20.83% and 38.10%, respectively (Table 1). Therefore, the application of the translational enhancer 6 × ARC not only amplify the phenotypic effects of *CpCYC1* and *CpCYC2* transgenes, but also increase the phenotype frequency under different genetic backgrounds, i.e., the wild-type plants and *cyc1 cyc2* double-mutant of *C. pumila*.

### 2.5. Phenotypes of AtMYB75 Transformed with the Fusion of 6 × ARC in A. thaliana

**Figure 5:**
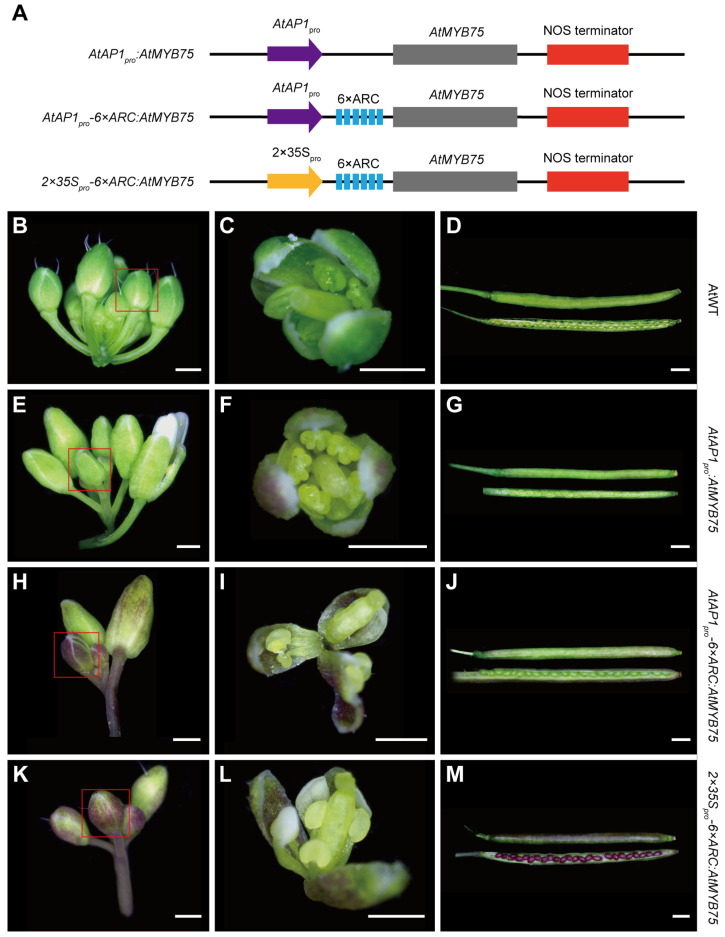
The flowers and fruits of *AtAP1_pro_:AtMYB75*, *AtAP1_pro_-6×ARC:AtMYB75*, and *2×35S-6×ARC:AtMYB75* transgenic plants of *A. thaliana* after cold treatment. (**A**) The structure of plasmids expressing *AtMYB75*. (**B**–**D**) The flowers and seeds of wild-type plants. (**E**–**G**) The flowers and seeds of *AtAP1_pro_:AtMYB75*. (**H**–**J**) The flowers and seeds of *AtAP1_pro_-6×ARC:AtMYB75*. (**K**–**M**) The flowers and seeds of *2×35S-6×ARC:AtMYB75*. The red rectangles indicate the flowers used for dissection. Scale bars: 0.5 mm.

In order to explore whether 6 × ARC can be applied to different plants, we constructed *AtAP1_pro_:AtMYB75*, *AtAP1_pro_-6×ARC:AtMYB75*, and *2×35S_pro_-6×ARC:AtMYB75* plasmids (Figure 5A), and carried out transgenic experiments in model plant *A. thaliana*. *AtMYB75* was selected as a target gene because its overexpression can cause the accumulation of anthocyanin pigments, leading to the purple pigmentation of multiple tissues, such as leaves, roots, stems, and sepals [36]. The *AtAP1* promoter was utilized to regulate the expression of *AtMYB75* as an expectation to produce purple flowers due to the fact that the *AtAP1* gene is mainly expressed in reproductive organs, such as floral meristems, young flower primordia, sepals, petals, and pedicels [41,42].

After 10 days of screening culture, we found that the epicotyl of *AtAP1_pro_-6×ARC:AtMYB75* transgenic lines showed obvious purple pigmentation, while the seedlings of *AtAP1_pro_:AtMYB75* transgenic lines with no 6 × ARC did not show any purple pigmentation, similar to wild-type plants (Figure S4A,B). In *2×35S_pro_-6×ARC:AtMYB75* transgenic lines, both the cotyledons and epicotyl were purple (Figure S4A,B). However, the purple pigmentation of *AtAP1_pro_-6×ARC:AtMYB75* and *2×35S_pro_-6×ARC:AtMYB75* occurred during the seedling stage but disappeared after transgenic seedlings were transferred to the soil and cultured at 20–22 °C (Figure S4C).

Previous research has shown that the anthocyanin accumulation is induced by abiotic stress, such as nitrogen or phosphate starvation, high sucrose levels, plant hormones, high light, and low temperature [36,43,44,45,46,47,48,49]. Therefore, we treated all transgenic and wild-type plants at a low temperature (15 °C) for 10 days. After the low-temperature treatment, the normal growth of *A. thaliana* was not significantly affected, and the color of different tissues of wild-type plants was not changed (Figure 5B–D and Figure S5A). However, purple pigmentation occurred in various transgenic plants at different degrees. Most tissues of *2×35S_pro_-6×ARC:AtMYB75* transgenic lines were dark purple, including leaves (Figure S5B,C), sepals, pedicels, inflorescence stems, pods, and seeds (Figure 5K–M). In contrast, *AtAP1_pro_-6×ARC:AtMYB75* transgenic lines exhibited intense purple pigmentation in specific tissues, i.e., sepals, pedicels, and inflorescence stems (Figure 5H,I), with weak purple pigmentation in the leaves and pods (Figure 5J and Figure S5B,C). In *AtAP1_pro_:AtMYB75* transgenic lines, only a small portion of the leaves and sepals turned to light purple (Figure 5E–G and Figure S5B,C). Taken together, the fusion of 6 × ARC to the 5′ end of the specific *AtAP1* promoter resulted in the overexpression of the target gene *AtMYB75*, with strong purple pigmentation in specific tissues or development stages in transgenic plants. Furthermore, the intensity of purple pigmentation in the sepals and pedicels of *AtAP1_pro_-6×ARC:AtMYB75* is comparable to that in *2×35S_pro_-6×ARC:AtMYB75*, clearly indicating that the joint action of the specific promoter and the translational enhancer 6 × ARC is effective to amplify the function of a gene in specific tissues or organs.

## 3. Discussion

Enhancing the expression of exogenous genes in transgenic plants is important for both gene functional analysis and plant genetic engineering. The application of translational enhancers can increase the translational level of exogenous genes without the modification of their spatiotemporal expression patterns. ARC is a sequence element that complements the 18S rRNA within the 40S ribosomal subunit and therefore increases the chance of mRNAs for translation [30]. However, whether ARC is applicable for transgenic research is not fully understood due to the scarcity of functional evidence. In this research, we have screened a high-efficient translational enhancer 6 × ARC by using two transient gene expression systems, i.e., *N. benthamiana* and *C. pumila*. We further evaluated the promising application value of this translational enhancer in gene function research by stable transgenic experiments in a new model plant *C. pumila* and the classical model system *A. thaliana*.

### 3.1. The High Efficiency of 6 × ARC in Amplifying the Phenotypic Effects of Exogenous Genes

To amplify the phenotype effects of exogenous genes in transgenic plants, strong promoters or translational enhancers are commonly utilized to improve their expression levels at the transcriptional or translational level, respectively. The widely used promoters are CaMV 35S promoter [5] and its duplicated form [6], maize polyubiquitin promoter [7], and rice actin promoter [8], while different 5′ UTR sequences from various plant virus RNA [17,18,19,20] or plant genes [25,26] are frequently applied as translational enhancers. In this study, through transient gene expression assays in *N. benthamiana* and *C. pumila*, we confirmed the high efficiency of 3 × ARC, which was first identified by [30], in enhancing the translational level of the reporter gene. We further generated six tandem repeats of ARC (6 × ARC) which demonstrated a powerful translational efficiency in *C. pumila* mesophyll protoplasts. Subsequently, we explored the application potential of 6 × ARC in gene functional research by detailed transgenic experiments in two model systems. Briefly, in *C. pumila*, the fusion of 6 × ARC greatly enhances the inhibiting effect of *CpCYC1* and *CpCYC2* genes on stamen development and yellow pigmentation in both wild-type plants and the *cyc1 cyc2* double-mutant. In *A. thaliana*, the application of 6 × ARC greatly promotes the function of *AtMYB75* in regulating purple pigmentation in sepals, pedicels, and inflorescence stems. Our results show that the translational enhancer 6 × ARC is highly efficient in enhancing the phenotype of exogenous genes in different plant species, with an effect obviously over the constitutive 2 × 35S promoter and the transcriptional activator domain VP16 derived from herpes simplex virus [50,51].

The binding of mRNA to the 43S pre-initiation complex is one of the rate-limiting steps in translation initiation [52]. ARC is a sequence element complementing an internal 18S rRNA segment of rice and can enhance the translational efficiency by increasing the cap-independent recruitment of 40S subunits [30,53]. Thus, the use of multiple copies of ARC sequences can significantly amplify the translational efficiency of mRNA by increasing the affinity for the 40S ribosomal subunits. Moreover, the region of 18S rRNA corresponding to ARC is highly conserved across rice, tobacco, maize, wheat, and human, suggesting that ARC may have broader applications than translational enhancers derived from plant virus RNA and plant genes [30]. Given the complementarity between ARC and 18s rRNA from monocotyledonous rice, we proceeded to investigate the translational efficiency of ARC in dicotyledonous plants. As expected, in this study, both transient gene expression assays and stable transgenic experiments demonstrated that 6 × ARC is highly efficient in amplifying the expected phenotypes in different plant systems, spanning the Asteridae to the Rosidae, indicating that it has an extensive application prospect. On the contrary, some of the currently used translational enhancers function well in some plant species but are unworkable in others. For example, the *AtADH 5′UTR* and *NtADH 5′UTR* enhance the translation of mRNA in *A. thaliana* and *N. benthamiana*, but fail in *O. sativa* [25]. Instead, the *OsADH* 5′UTR works in *A. thaliana*, *N. benthamiana*, and *O. sativa* [25]. Furthermore, the translational enhancers found in plant virus TMV and TEV are effective in eudicots, but inefficient in monocots [28]. In addition, our results demonstrate that the use of 6 × ARC in transgenic plants not only produces a strong expected phenotype but also significantly increase the phenotype frequency by greatly promoting the expression of exogenous genes in transgenic plants. By using 6 × ARC in transgenic plants, the frequency of the expected phenotype is averagely over 50%, thus greatly saving time and cost in transgenic experiments.

### 3.2. Advantage of 6 × ARC in Manifesting the Function of Exogenous Genes in Transgenic Plants

As pivotal regulator factors for floral symmetry, *CYC*-like genes have been known to not only determine the identity of dorsal floral organs in the second and third whorls, but also control dorsoventrally asymmetric floral color patterning [33,35,54]. For example, in *Torenia fournieri* (Linderniaceae), the overexpression of either *TfCYC1* or *TfCYC2* under the control of 35S promoter results in a loss of yellow spots from the ventral petal [54]. *TfCYC* genes, especially *TfCYC2*, have the potential to establish the bilateral petal pigmentation in *T. fournieri* because they can bind directly to the regulatory regions of the R2R3-MYB gene *TfMYB1* [54]. However, in *TfCYC2*-RNAi transgenic plants with down-regulated *TfCYC2* activity, the violet pigmentation extends from two lateral petals to two dorsal petals, but the yellow spots are still restricted to the ventral petal rather than expanding into other floral regions. Therefore, the function of *TfCYC2* in repressing yellow pigmentation awaits further functional verification. In naturally occurring *peloric* flower varieties of *Primulina heterotricha* and *C. pumila*, both belonging to the Gesneriaceae family, the expression signals of *CYC*-like genes are completely lost, consistent with the extended yellow spots over the corolla tube [33,55]. In addition, the overexpression of either *CpCYC1* or *CpCYC2* under the control of the duplicated CaMV 35S promoter generates dorsalized actinomorphic flowers accompanied by the loss of yellow spots, while the double-mutation of these two genes has the phenotypes of ventralized actinomorphic flowers with an extension of yellow pigmentation across corolla tube [35]. In addition, a previous study shows that *CpCYC1* and *CpCYC2* genes can inhibit the formation of yellow spots out of the ventral region of the flower by directly repressing *CpF3′5′H* [35]. However, when *CpF3′5′H* is silenced by virus-induced gene silencing (VIGS), the yellow spots in both the wild-type plants and *cyc1 cyc2* double-mutant are only weakened [35], implying that other genes or regulatory pathways might also be involved in controlling the yellow pigmentation. As outlined above, no conclusive evidence in transgenic functional analyses shows that *CYC*-like genes have the complete function of repressing the yellow pigmentation [35,54].

In this study, the transformations of *CpCYC1_pro_:CpCYC1*, *CpCYC2_pro_:CpCYC2*, and *CpCYC2_pro_:CpCYC2-VP16* plasmids into the *cyc1 cyc2* double-mutant of *C. pumila* produce local distribution of yellow spots near the throat of the corolla tube. Nevertheless, yellow spots are still clearly visible in these transgenic lines. On the contrary, the transformation of either *CpCYC1_pro_-6×ARC:CpCYC1* or *CpCYC2_pro_-6×ARC:CpCYC2* generates phenotypes with a complete loss of yellow pigmentation from the corolla tube. Therefore, our results provide strong evidence that *CYC*-like genes in *C. pumila* (*CpCYC1/2*) may have complete function of the negative control of the yellow pigmentation. Given that the proteins of *CYC*-like genes can bind directly to the regulatory regions of transcription factor R2R3-MYB genes and flavonoid synthesis-related genes [35,54], the genes involved in the control of corolla pigmentations might be downstream-targeted by CYC-like proteins.

Transcription factors usually serve two essential functions: binding the appropriate DNA contact sites in their target genes and recruiting other proteins to execute transcriptional control. The binding activity in an individual DNA site usually involves protein complexes with proteins co-opted or competed [56]. For an individual DNA-binding protein in relation to given DNA sites, the protein–DNA interaction includes specific and non-specific binding activities. The non-specific binding is usually highly flexible in contrast to the rigid interface in the case of specific binding, thus implicating dynamics in specificity [56,57]. In addition, according to [30], the ARC sequence which complements an internal 18S rRNA segment of rice artificially introduced into a mRNA leader can considerably increase the cap-independent recruitment of 40S subunits and mRNA translation efficiency. Two or more copies of ARC in the leader have a considerably higher effect than a single copy [30]. Accordingly, the activities of *CYC*-like genes in *C. pumila* much enhanced by 6 × ARC may enable their proteins to bind to both specific and non-specific DNA-binding sites of all their downstream target genes; otherwise, it can only bind to the specific sites of their target genes. In addition, the expressions of *CpCYC1/2* significantly raised by 6 × ARC should make their proteins achieve great advantage in competition over other proteins in the protein-binding complex. These may be the main reason why the genetic transformations of *CpCYC1_pro_-6×ARC:CpCYC1* and *CpCYC2_pro_-6×ARC:CpCYC2* generate phenotypes with a complete loss of yellow pigmentation, while yellow pigmentations are only weakened or reduced from the corolla tube under other transgenic conditions. Thus, the translational enhancer ARC with six tandem repeats, i.e., 6 × ARC, has great advantage in manifesting the function of exogenous genes in transgenic plants.

## 4. Materials and Methods

### 4.1. Plant Materials and Growth Conditions

The *C. pumila* D. Don (Wang, HK01) used in this study was collected from Hekou County, Yunnan, China. The plants were grown in 7 cm pots containing a mixture of vermiculite and commercially available humus soil (1:2) in a growth chamber using the following controlled conditions: 10 h light (25 °C) and 14 h dark (21 °C) photoperiods, and 50–70% relative humidity. For transient gene expression assays and transgenic experiments, the seeds were surface-sterilized and germinated on a half-strength Murashige and Skoog (1/2 MS) medium supplemented with 10 g/L sucrose and 0.02 mg/L α-naphthlcetic acid [32,33]. About one month later, the seedlings were transferred to 1/2 MS medium supplemented with 20 g/L sucrose. Tissue culture was conducted in a growth chamber with 12 h light and 12 h dark photoperiods at 25 °C.

*N. benthamiana* seeds were germinated in 7 cm pots containing a mixture of vermiculite and commercially available humus soil (1:2). After two weeks, the seedlings were transferred to new pots and grown in a growth chamber with 16 h light (23 °C) and 8 h dark (20 °C). About 1.5-month-old plants were used for transient gene expression assays. *A. thaliana* (ecotype Columbia-0) seeds were germinated on a 1/2 MS medium containing 30 g/L sucrose. The seedlings were transplanted into the soil and grown to the flowering stage under 16 h light (23 °C) and 8 h dark (21 °C) conditions.

### 4.2. DNA and RNA Isolation

Genomic DNA (gDNA) was extracted from the fresh leaves of *C. pumila*, *Petrocosmea minor*, and *A. thaliana*. Total RNA was extracted from the young floral buds of *C. pumila* and *A. thaliana* using an SV Total RNA Isolation System (Promega, Madison, WI, USA), following the manufacturer’s instructions. Complementary DNA (cDNA) was synthesized using the RevertAid H Minus First-Strand cDNA Synthesis Kit (Thermo Scientific, Waltham, MA, USA).

### 4.3. Transient Gene Expression Assays

The promoter of *PmCIN1* (2069 bp, *PmCIN1*_pro_) was amplified from *P. minor* and cloned into the *BamH* I and *Sac* II sites of the *pGreenII 0800-LUC* vector to obtain the *PmCIN1_pro_:LUC* plasmid using the In-Fusion HD Cloning Kit (TaKaRa, Dalian, China). Translational enhancers Ω, *OsADH* 5′ UTR (*ADH* for short), 3 × ARC, 4 × ARC, 5 × ARC, and 6 × ARC were inserted between the *PmCIN1_pro_* and *LUC* of the *PmCIN1_pro_:LUC* plasmid to obtain six reporter plasmids, including *PmCIN1_pro_-Ω:LUC*, *PmCIN1_pro_-ADH:LUC*, *PmCIN1_pro_-3×ARC:LUC*, *PmCIN1_pro_-4×ARC:LUC*, *PmCIN1_pro_-5×ARC:LUC*, and *PmCIN1_pro_-6×ARC:LUC*. The reporter plasmids with different translational enhancers were, respectively, transformed into *C. pumila* mesophyll protoplasts, as described previously [34]. The *PmCIN1_pro_:LUC* plasmid was transformed as the control. Each transformation experiment included three independent biological replicates.

For the transient gene expression assay using *N. benthamiana* leaves, reporter plasmids, including the control *PmCIN1_pro_:LUC*, were, respectively, transformed into *Agrobacterium tumefaciens* GV3101 pSoup strain. A single colony of GV3101 pSoup containing each plasmid was cultured in 1 mL of a YEB medium (containing 50 mg/L kanamycin and 50 mg/L rifampicin) with shaking (200 rpm) at 28 °C overnight. The next day, 0.5 mL of overnight cultures was inoculated into 5 mL of a fresh YEB medium and grown at 28 °C for 2–3 h. The cells were harvested by centrifuging at 5000 rpm for 5 min, rinsed with an MMA induction medium (10 mM MgCl_2_, 10 mM MES, 200 μM acetosyringone, pH 5.6), and finally resuspended in MMA to a final concentration of OD600 = 0.6. The bacteria suspensions were infiltrated into young and fully expanded leaves using a needleless syringe. Each plasmid infiltrated at least three leaves. After infiltration, plants were immediately covered with plastic bags and incubated under normal growth conditions for 48 h.

Protein isolation and luciferase activity measurement were performed using the Dual-Luciferase Reporter Assay System (Promega, Madison, WI, USA), according to the manufacturer’s instructions. The relative luciferase activity was calculated as the ratio between LUC and RLUC. The data shown were calculated from at least three independent biological replicates.

### 4.4. Genetic Transformation of C. pumila

1439 bp (1.4 Kb) of *CpCYC1* promoter and 1747 bp (1.7 Kb) of *CpCYC2* promoter [58] were amplified from the *C. pumila* gDNA sample. PCR products were purified and cloned into the *EcoR* I and *Pst* I sites of *pCAMBIA1301* vector using the In-Fusion HD Cloning Kit (TaKaRa, Dalian, China) to obtain *1301-CpCYC1_pro_* and *1301-CpCYC2_pro_* plasmids. The full-length coding sequences (CDSs) of *CpCYC1* and *CpCYC2* were amplified from *C. pumila* cDNA and, respectively, inserted into the *Pst* I and *BstP* I sites of *1301-CpCYC1_pro_* and *1301-CpCYC2_pro_* plasmids to obtain *CpCYC1_pro_:CpCYC1* and *CpCYC2_pro_:CpCYC2* plasmids. To construct plasmids harboring the translational enhancer 6 × ARC, the 6 × ARC element was amplified and cloned into the *Pst* I and *Nco* I sites of the *pCAMBIA1301* vector to obtain the *1301-6×ARC* plasmid. *CpCYC1* and *CpCYC2* promoters were then cloned into the *EcoR* I and *Pst* I sites of the *1301-6×ARC* plasmid to obtain *CpCYC1_pro_-6×ARC* and *CpCYC2_pro_-6×ARC* plasmids. The full-length CDSs of *CpCYC1* and *CpCYC2* were finally inserted into the *Nco* I and *BstP* I sites of *CpCYC1_pro_-6×ARC* and *CpCYC2_pro_-6×ARC* plasmids to obtain *CpCYC1_pro_-6×ARC:CpCYC1* and *CpCYC2_pro_-6×ARC:CpCYC2* plasmids. To construct plasmids carrying the transcriptional activation domain VP16 [59], the full-length CDSs of *CpCYC1* and *CpCYC2* were fused in-frame with the VP16 domain to generate *CpCYC1_pro_:CpCYC1-VP16* and *CpCYC2_pro_:CpCYC2-VP16* plasmids. All plasmids were, respectively, introduced into *A. tumefaciens* strain LBA4404 and transformed into the wild-type *C. pumila* plants and *cyc1 cyc2* double-mutant constructed by [35] as described previously [32,33]. The transgenic plants were confirmed by PCR using genomic DNA, as templates. All primers used for vector construction are listed in Table S1.

### 4.5. Genetic Transformation of A. thaliana

The full-length CDS of *AtMYB75* was amplified and cloned into the *Hind* III and *Nco* I sites of *pCAMBIA1302* to obtain *1302-AtMYB75* plasmid. Then, 1758 bp (1.7 Kb) of *AtAP1* promoter [60] was amplified from the *A. thaliana* gDNA sample and cloned into the *Kpn* I and *Hind* III sites of *1302-AtMYB75* plasmid to obtain *AtAP1_pro_:AtMYB75* plasmid. To construct plasmids harboring the translational enhancer 6 × ARC, the 6 × ARC element and full-length CDS of *AtMYB75* were inserted into the *Sal* I and *Nco* I sites of *pCAMBIA1302* to obtain *6×ARC:AtMYB75* plasmid using the In-Fusion HD Cloning Kit (TaKaRa, Dalian, China). *AtAP1* promoter was then cloned into the *Kpn* I and *Sal* I sites of *6×ARC:AtMYB75* plasmid to obtain *AtAP1_pro_-6×ARC:AtMYB75* plasmid. The duplicated CaMV 35S promoter was amplified and inserted into the *EcoR* I and *Sal* I sites of *6×ARC:AtMYB75* plasmid to obtain *2×35S_pro_-6×ARC:AtMYB75* plasmid. All plasmids were introduced into *A. tumefaciens* strain EHA105 and transformed into *A. thaliana,* as described previously [61]. All primers used for vector construction are listed in Table S1.

### 4.6. Real-Time Quantitative PCR (RT-qPCR) Assays

For the RT-qPCR expression analysis of *CpCYC1* and *CpCYC2* genes, dorsal, lateral, and ventral petals were harvested from about 1.5 cm flower buds of wild-type, *cyc1 cyc2* double-mutant, and transgenic plants. Each sample included three biological replicates. Total RNA was extracted and cDNA was synthesized as described above. RT-qPCR was performed using the TB Green Premix Ex Taq (TaKaRa, Dalian, China) with ROX as a reference dye on a StepOne Plus Real-time PCR System. The PCR conditions were as follows: initial denaturation at 95 °C for 30 s, 40 cycles of 95 °C for 5 s, and 60 °C for 30 s. Dissociation curves were recorded using 1 cycle of 95 °C for 15 s, 60 °C for 60 s, and 95 °C for 15 s. The relative expression level of each gene was determined using the 2^−ΔCT^ method [62] and *CpACTIN* was used as the internal control. For the RT-qPCR expression analysis of *LUC* gene, the *C. pumila* mesophyll protoplasts which transformed the reporter plasmids *PmCIN1pro:LUC* (control) and *PmCIN1pro-6×ARC:LUC* were harvested. The *RLUC* gene was used as the internal control for protoplasts resulting from the transient expression analysis. The primers used for RT-qPCR are listed in Table S1.

## 5. Conclusions

In summary, we conducted a comprehensively comparative research on the translational enhancer ARC within both transient expression and agrobacterium-mediated transformation systems. We found that the ARC with six tandem repeats, i.e., 6 × ARC, exhibits great translational efficiency, over 130 times above the translation level without the insertion of any enhancer within the transient expression system. In agrobacterium-mediated transformation systems, 6 × ARC displays a great power to amplify the phenotypic effect of exogenous genes, with advantage over the constitutive 2 × 35S promoter and the transcriptional activator domain VP16 in transgenic plants. In *C. pumila*, the fusion of 6 × ARC significantly promotes the repressing effect of *CpCYC1* and *CpCYC2* genes on stamen development and yellow pigmentation. In *A. thaliana*, the application of 6 × ARC greatly enhances the effect of *AtMYB75* in controlling purple pigmentation in sepals, pedicels, and inflorescence stems. Most significantly, the fusion of 6 × ARC in *CpCYC1/2* transgenic system can enable us to fully reveal that *CYC*-like genes in *C. pumila* have the complete function of repressing the yellow spots by broadly negatively controlling its downstream factors or elements involved in color pigmentations. For manifesting the function of exogenous genes, apparently, the 6 × ARC displays a big advantage over 2 × 35S promoter and the transcriptional activator domain VP16. It would be appreciated to conform its translational efficiency in broad ranges of taxa and make use of it as a fundamental tool in genetic transformation, as well as in the agronomic practices of crop engineering.

## Figures and Tables

**Table 1:** The effect of 6 × ARC on the phenotype frequency of *CpCYC1/2* transgenes in *C. pumila*.

Transgenic Background	Plasmid	The Number of Positive Transgenic Plants	The Number of Transgenic Plants with Phenotype	Phenotype Frequency (%)
WT	*CpCYC1_Pro_:CpCYC1*	18	5	27.78
WT	*CpCYC1_Pro_-6×ARC:CpCYC1*	32	21	65.63
WT	*CpCYC1_Pro_:CpCYC1-VP16*	22	8	36.36
*cyc1 cyc2*	*CpCYC1_Pro_:CpCYC1*	21	6	28.57
*cyc1 cyc2*	*CpCYC1_Pro_-6×ARC:CpCYC1*	27	14	51.85
*cyc1 cyc2*	*CpCYC2_Pro_:CpCYC2*	24	5	20.83
*cyc1 cyc2*	*CpCYC2_Pro_-6×ARC:CpCYC2*	24	12	50.00
*cyc1 cyc2*	*CpCYC2_Pro_:CpCYC2-VP16*	21	8	38.10

## Data Availability

Source data are provided in this paper. All additional data that support the findings of this study are available from the corresponding author upon reasonable request.

## Supplementary Materials

The following supporting information can be downloaded at: https://www.mdpi.com/article/10.3390/plants13223120/s1, Figure S1: The sequences of translational enhancers Ω, *ADH*, 3 × ARC, 4 × ARC, 5 × ARC, and 6 × ARC; Figure S2: Real-time qPCR expression analysis of *LUC* between *PmCIN1pro:LUC* (control) and *PmCIN1pro-6×ARC:LUC*. Figure S3: Real-time qPCR expression analysis of *CpCYC1* in *CpCYC1_pro_:CpCYC1*, *CpCYC1_pro_-6×ARC:CpCYC1*, and *CpCYC1_pro_:CpCYC1-VP16* transgenic plants; Figure S4: The phenotype of wild-type, *AtAP1_pro_:AtMYB75*, *AtAP1_pro_-6×ARC:AtMYB75*, and *2×35S-6×ARC:AtMYB75* transgenic plants of *A. thaliana* before cold treatment; Figure S5: The transformation of two constructs carrying 6 × ARC generated purple leaves in *A. thaliana* plants after cold treatment. Table S1: All primers used for vector construction and RT-qPCR.
