# Supplementary material for: Application of an Efficient Enhancer in Gene Function Research
Source: Plants (Basel). 2024 Nov 6;13(22):3120. doi: 10.3390/plants13223120 (PMC11597595; doi:10.3390/plants13223120)
Supplement: Supplementary file 1 [file plants-13-03120-s001.zip › Supplementary Figures S1-S5.pdf]

Supplementary Information for

Application of an Efficient Enhancer in Gene Function Research

Feng-Xian Guo [1,2,3,†], Rui-Xue Yang [1,2,3,†], Xia Yang [1,2,†], Jing Liu [1,2] and Yin-Zheng Wang [1,2,3,*]

[1] State Key Laboratory of Plant Diversity and Specialty Crops and Key Laboratory of Systematic and Evolutionary Botany, Institute of Botany, Chinese Academy of Sciences, Beijing 100093, China; guofengxian7@163.com (F.-X.G.); yangrx_98@163.com (R.-X.Y.); yangxia@ibcas.ac.cn (X.Y.); liujing2010@ibcas.ac.cn (J.L.)
[2] China National Botanical Garden, Beijing 100093, China
[3] University of Chinese Academy of Sciences, Beijing 100049, China
* Correspondence: wangyz@ibcas.ac.cn
† These authors contributed equally to this work.

Ω (5’UTR of TMV RNA)

5’-TATTTTTACAACAATTACCAACAACAACAAACAACAAACAACATTACAATTACTATTTACAATTACA-3’

***ADH* (5’UTR of *OsADH*)**

5’-TACATCACAATCACACAAAACTAACAAAAGATCAAAAGCAAGTTCTTCACTGTTGATA-3’

3×ARC-1

5’- GCCTAAGCTTACAA**ATACTCCCCC**ACAACAGCTTACA**ATACTCCCCC**ACACAGCTTACAA**ATACTCCCCC**
ACAACAGCTTGTCGA-3’

4×ARC-1

5’- GCCTAAGCTTACA**ATACTCCCCC**ACACAGCTTACAA**ATACTCCCCC**ACAACAGCTTACA**ATACTCCCCC**A
CACAGCTTACAA**ATACTCCCCC**ACAACAGCTTGTCGA-3’

5×ARC-1

5’-GCCTAAGCTTACAA**ATACTCCCCC**ACAACAGCTTACA**ATACTCCCCC**ACACAGCTTACAA**ATACTCCCCC**A
CAACAGCTTACA**ATACTCCCCC**ACACAGCTTACAA**ATACTCCCCC**ACAACAGCTTGTCGA-3’

6×ARC-1

5’- GCCTAAGCTTACA**ATACTCCCCC**ACACAGCTTACAA**ATACTCCCCC**ACAACAGCTTACA**ATACTCCCCC**A
CACAGCTTACAA**ATACTCCCCC**ACAACAGCTTACA**ATACTCCCCC**ACACAGCTTACAA**ATACTCCCCC**ACAA
CAGCTTGTCGA-3’

Figure S1. The sequences of translational enhancers Ω, *ADH*, 3 × ARC, 4 × ARC, 5 × ARC, and 6 × ARC. The 10 bp ARC are red color. Ω, 5’UTR of tobacco mosaic virus (TMV) RNA. *ADH*, 5’-UTR of the alcohol dehydrogenase gene from *O. sativa*. ARC, active ribosomal RNA complementary.

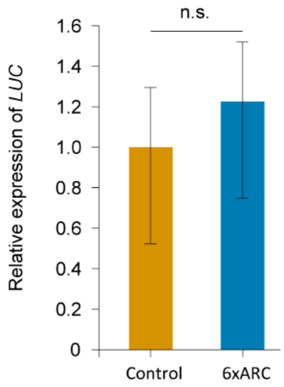

Figure S2. Real-time qPCR expression analysis of *LUC* between *PmCIN1pro:LUC* (control) and *PmCIN1pro-6×ARC:LUC*. The values shown are average of three biological replicates. The expression level of LUC in *PmCIN1pro-6×ARC:LUC* was normalized to the control. (Student's t-test, n.s. indicate non-significant, $P>0.05$).

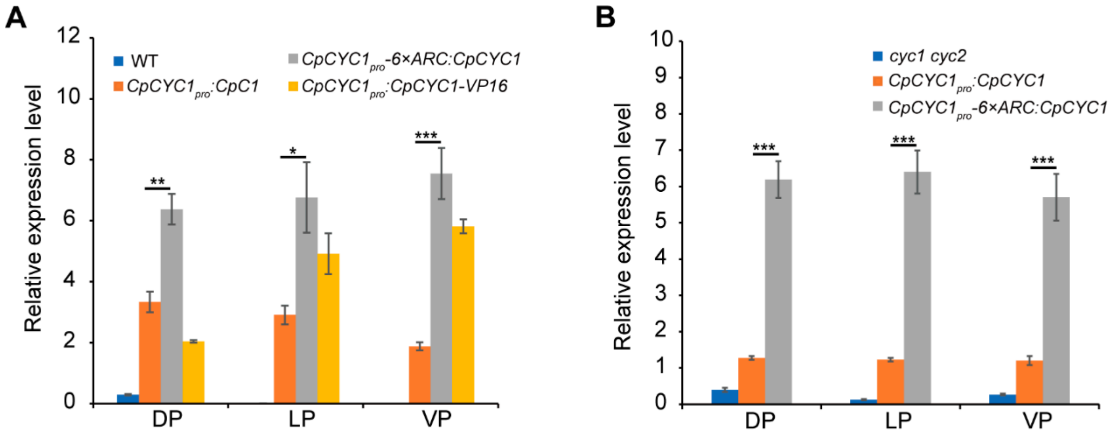

Figure S3. Real-time qPCR expression analysis of *CpCYC1* in *CpCYC1pro:CpCYC1*, *CpCYC1pro-6×ARC:CpCYC1*, and *CpCYC1pro:CpCYC1-VP16* transgenic plants. **(A)** Expression patterns of *CpCYC1* in transgenic plants under the wild-type background. **(B)** Expression patterns of *CpCYC1* in transgenic plants under the *cyc1 cyc2* double mutant background. The expression levels were normalized to those of *CpACTIN*. The error bars indicate the SD from 3 independent samples. DP, dorsal petals; LP, lateral petals; VP, ventral petals. Asterisks indicate significant differences between samples (Student's t-test, $^{*}P < 0.05$, $^{**}P < 0.01$, $^{***}P < 0.001$).

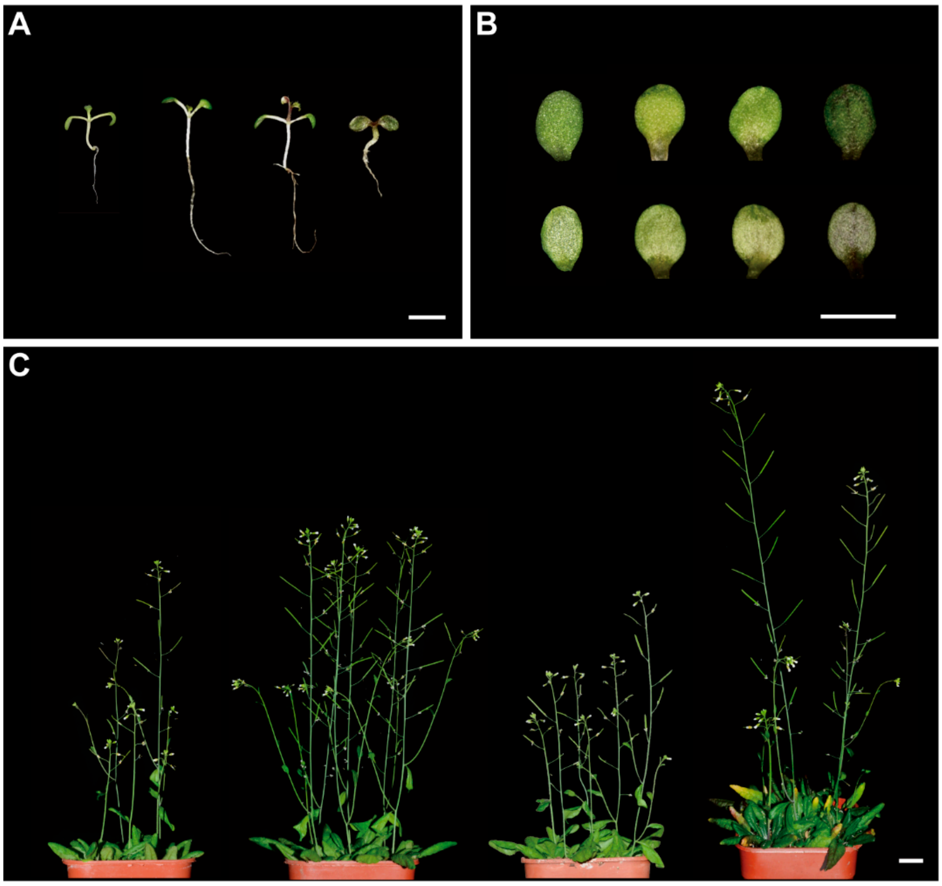

Figure S4. The phenotype of wild-type, *AtAP1$_{pro}$:AtMYB75*, *AtAP1$_{pro}$-6×ARC:AtMYB75*, and *2×35S-6×ARC:AtMYB75* transgenic plants of *A. thaliana* before cold treatment. **(A)** The seedlings of wild-type, *AtAP1$_{pro}$:AtMYB75*, *AtAP1$_{pro}$-6×ARC:AtMYB75*, and *2×35S-6×ARC:AtMYB75* from left to right. Scale bars: 5 mm. **(B)** The cotyledons of plants in figure A. Scale bars: 2.5 mm. **(C)** Wild-type and transgenic plants in figure A from left to right. Scale bars: 1 cm.

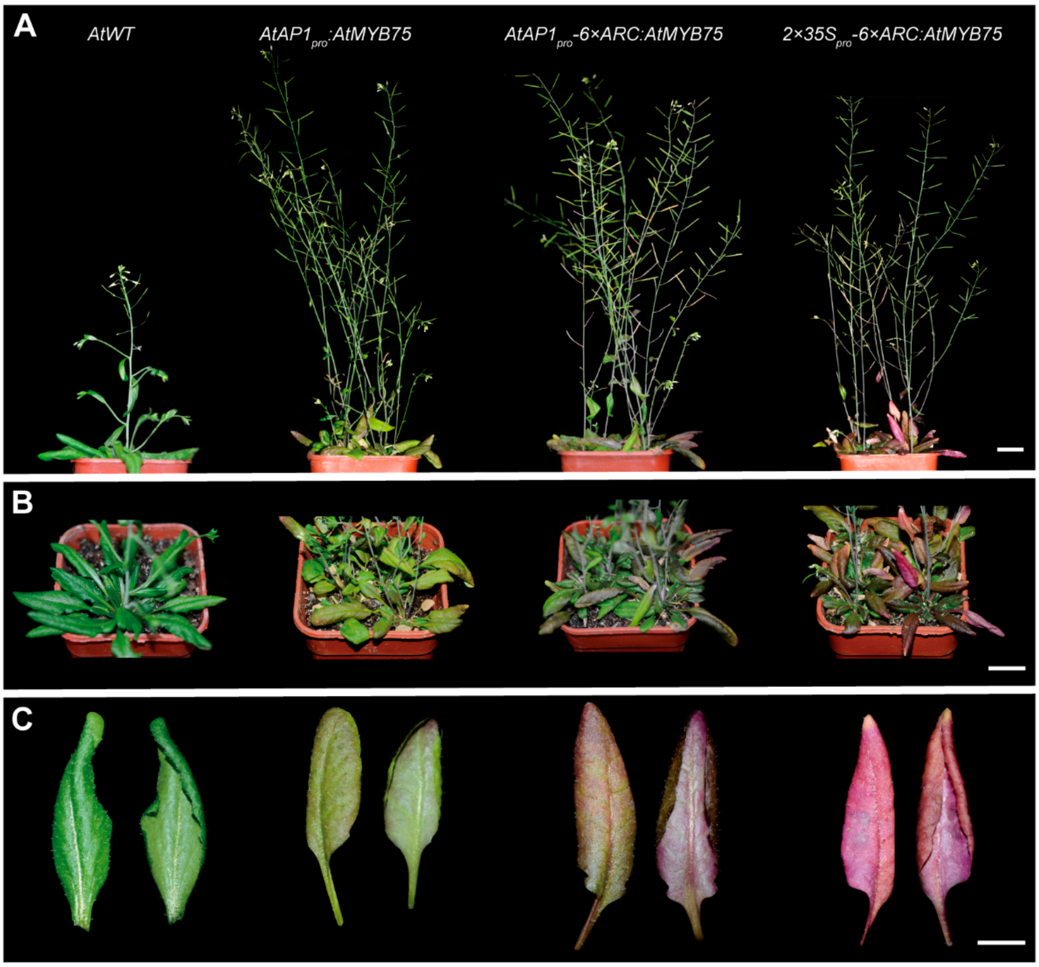

Figure S5. The transformation of two constructs carrying 6 × ARC generated purple leaves in *A. thaliana* plants after cold treatment. **(A and B)** The photos of wild-type, *AtAP1pro:AtMYB75*, *AtAP1pro-6×ARC:AtMYB75*, and *2×35S-6×ARC:AtMYB75* plants from left to right. **(C)** The adaxial and abaxial side of leaves corresponding to the transgenic plants in figure A. Scale bars: 1 cm.
